# Health Literacy Development among People with Chronic Diseases: Advancing the State of the Art and Learning from International Practices

**DOI:** 10.3390/ijerph19127315

**Published:** 2022-06-14

**Authors:** Jonas Lander, Marie-Luise Dierks, Melanie Hawkins

**Affiliations:** 1 Institute for Epidemiology, Social Medicine and Health Systems Research, Hannover Medical School, 30625 Hannover, Germany; dierks.marie-luise@mh-hannover.de; 2 Centre for Global Health and Equity, Faculty of Health, Arts and Design, Department of Health and Biostatistics, Swinburne University of Technology, Melbourne, VIC 3122, Australia; melaniehawkins@swin.edu.au

## 1. Introduction

Chronic diseases account for a considerable part of the strain on health care systems [1]. They are also burdensome for each affected individual and their families. In recent years, the concept of health literacy has been substantially elaborated on, particularly regarding the development and implementation of interventions at different levels, efforts to improve its measurement, and the role of communities and organizations. While a range of advancements are uncontested, specific challenges still revolve around: Thoroughly implementing modern practices of health literacy that do not focus on individual deficits but societal support of health literacy strengths and response to health literacy challenges [2]; developing, testing, and evaluating strategies for organizational health literacy responsiveness [3]; understanding the impact of eHealth literacy on health outcomes [4]; improving the co-design, local ownership, and integration of health literacy actions and interventions in communities experiencing vulnerability and disadvantage [5,6,7]; further refining measurement instruments, e.g., with less focus on self-assessments [8]; and addressing current health literacy support strategies by healthcare professionals [9].

This Special Issue was open to submissions about research addressing these aspects and more about advancing health literacy, and had a specific focus on developing health literacy among people with chronic diseases. Health literacy development is about advancements in health practices, organizations, and policies that create enabling environments in which people have the necessary knowledge and feel confident and comfortable accessing, understanding, and using health information and services. Enabling environments are especially necessary for people who are managing health conditions (often more than one condition) over long periods of time, more so for people experiencing vulnerability and disadvantage. This Special Issue includes 10 articles from five countries, which we discuss here according to the distinct perspectives from which health literacy might be approached or developed: individual, professional, and organizational.

## 2. Articles in the Special Issue

### 2.1. Health Literacy Development as an Individuals’ Effort and/or as a Combined Effort between Target Populations and Professionals

In “Health Literacy Co-Design in a Low Resource Setting: Harnessing Local Wisdom to Inform Interventions across Fishing Villages in Egypt to Improve Health and Equity”, Anwar et al. [5] describe how researchers and a local fishing community used the Ophelia (Optimising Health Literacy and Access) process [10] to co-design ideas for health literacy interventions in a low-resource setting. The article highlights that health literacy development is likely to be more effective and feasible if a community identifies its own diverse health literacy strengths, needs, and preferences and has local ownership of the actions that are subsequently integrated into intervention planning. Unfortunately, this type of genuine co-design of health literacy interventions seems to be an exception rather than a standard. The authors also point out the need for focusing on groups and communities experiencing disadvantage who may be more difficult to reach but who benefit most from appropriate, meaningful, and useful approaches for strengthening health literacy.

The cross-sectional survey study “Electronic Health Literacy in Individuals with Chronic Pain and its Association with Psychological Function” by Castarlenas et al. [11] examines the association between electronic health literacy (eHealth literacy) and health-related behaviours in people with chronic pain, not least because people with chronic illnesses make more frequent use of and rely on health information and services more than individuals without such conditions. According to the authors, the “good news” for clinical implications is that eHealth literacy can be learned, which is important for (electronic) health literacy development given the challenges that people have in finding good quality health information in the seemingly endless online information landscape. With respect to the special role, i.e., the importance of navigational health literacy and hence, strategies to improve navigation skills among individuals, needs not to be forgotten, given the number of recent studies pointing at challenges for individuals when trying to find targeted and information landscape, particularly in online environments. 

“How Can Cardiac Rehabilitation Promote Health Literacy? Results from a Qualitative Study in Cardiac Inpatients” by Isselhard et al. [12] contributes to the exploration and discussion of relevant, already existing, and “hidden”, i.e., overlooked domains of the current concept and understanding of health literacy. The empirical investigation exemplifies the importance of integrating patient perspectives into health literacy conceptualizations. This is, on the one hand, to “approve” or consent to experts and healthcare professionals’ accounts and understandings about health literacy, and on the other hand, to include patients’ perspectives of health literacy, which is essential to revealing potentially important (new) components of the concept that would be otherwise invisible. The example of cardiac rehabilitation highlights that different target groups may place different emphasis on single components of health literacy, which supports, for instance, Anwar et al.’s argument against one-size-fits-all approaches.

In the mixed-methods study “Preferences and Experiences of People with Chronic Illness in Using Different Sources of Health Information” by Gille et al. [13], the authors investigate information-seeking behaviour and information preferences in a generic sample of chronically ill persons. The findings are valuable for the discussion about health literacy development in so far as, despite the wide availability of digital health information, chronically ill people still consider doctors and other healthcare professionals to be the most useful and trusted source of information. While calls for the role and responsibility of healthcare professionals as, for example, “health literacy mediators” [14] seem obvious, healthcare professionals’ first and foremost (or traditional?) responsibility lies in providing diagnosis and treatment and, where relevant, prevention. The challenge for health literacy development may therefore lie in identifying what could be called “windows of opportunities” within established healthcare structures to promote feasible and resource-friendly health literacy interventions.

### 2.2. Health Literacy Development through Healthcare Professions

Budhatoki et al. [15] provide an argument for valid health literacy measurements as the foundation for any evidence-based approach to identifying health literacy needs, and based on that, developing suitable interventions in their validity testing study “Use of the English Health Literacy Questionnaire (HLQ) with Health Science University Students in Nepal”. As the case of Nepali health science students illustrates, identifying the health literacy of (future) healthcare professionals is so important because only when individuals working in healthcare institutions are aware of their own health literacy resources can they better understand how to meaningfully support the health literacy of the people they serve. In terms of health literacy development, the authors also forward an argument for including health literacy training early in academic and medical education.

Combining a cross-sectional and longitudinal study design, Voigt-Barbarowicz and colleagues [16] asked in-clinic rehabilitation patients to estimate their health literacy (at the start and at the end of their rehabilitation stay) and compared the results with a health literacy estimation of the same patients by their respective treatment providers, i.e., physicians, physiotherapists, nurses, and social workers. Regarding health literacy development, one important finding of “Patients’ Health Literacy in Rehabilitation: Comparison between the Estimation of Patients and Health Care Professionals” is that—in this study—initial improvements in patient’s health literacy are difficult to maintain months after treatment termination, causing the authors to call for a more sustained way of fostering individual health literacy, for instance, in the phase after care. The other, maybe even more important, finding relates to the fact that healthcare professionals in this study overestimate their patients’ health literacy—the comparison indicates only poor to fair agreements in accordance with previous research findings [17,18,19]. This has important implications for approaches to develop and strengthen health literacy in individuals because healthcare provider-related efforts to do so may only be targeted, reasonable, and meaningful once health literacy is accurately estimated by respective professionals.

In “Health Literacy-Sensitive Counselling on Early Childhood Allergy Prevention: Results of a Qualitative Study on German Midwives’ Perspectives”, von Sommoggy et al. [20] showcase concrete examples of how healthcare professionals—here, midwives—convey health literacy-related knowledge and competencies towards their clients. One encouraging point made by this study—based on the perspectives of interviewed midwives—is that there are actual “windows of opportunity”, such as the phase of pregnancy, that seem particularly well suited to develop and strengthen health literacy in respective target populations. For example, understanding and applying effective early allergy prevention measures as a parent, using evidence-based health information. However, von Sommoggy and colleagues find that midwives do not explicitly counsel in a health literacy-sensitive way. That is, they do not emphasize health literacy as a standalone topic but usually only implicitly convey knowledge and competencies, e.g., regarding awareness about available allergy prevention guidelines. As such, the study provides another argument for focusing healthcare professionals’ attention on health literacy, and according to the authors, this ideally happens already during the formal qualification and training phase.

### 2.3. Health Literacy Development through Healthcare Organizations

The importance of developing health literacy research and practice from the patients’ perspective is revealed in “Organizational Health Literacy in a Hospital—Insights on the Patients’ Perspective” by Lubasch et al. [21]. The authors investigate potential associations between individual patient characteristics, their perceptions, and health-literacy-sensitive communication by hospital staff and the respective organizational structures underlying such communication. One of the central findings, namely the strong association between a hospital’s organization and health-literacy-sensitive communication towards patients, not only supports the argument for understanding health literacy as a relations construct between individuals and organizations—emphasizing institutional and organizational responsibility to create health-literate environments. Moreover, and in line with the arguments provided by, e.g., Anwar et al. [5] and Isselhard et al. [12], it may also be seen as a good and feasible opportunity for healthcare organizations struggling to create health-literacy-sensitive structures: target group perspectives on what constitutes a health literate organization may be used as starting point for any organizational, structural, or cultural change.

As organizational health literacy seems to lie in the hands of healthcare institutions and the professionals working within these institutions, Meldgaard et al. [22] outlined how (lay) target group perspectives may be integrated into processes that aim to foster individual HL through organizational efforts. Their study protocol reports on the intended use of the Ophelia process (see above) to develop an antenatal care intervention, which, in turn, is intended to foster pregnant women’s health literacy. Since pregnant women are in a specific phase of life, this needs to be accounted for when adapting health information services to their needs and preferences. Importantly, the authors do not only point to the various potential benefits of applying the Ophelia process towards health literacy development efforts. Moreover, they refer to the conditions and prerequisites required to make health literacy co-design approaches work, not least in terms of its’ participants’ confidence and capabilities to engage effectively in in-depth cooperation with researchers and vice versa.

Lastly, as shown by Huebner et al. [23], health literacy development will be increasingly related to ethical aspects, such as the dependence on technology to make “good” health decisions or the extent of actual responsibility of health professions and healthcare systems to support individuals in becoming health literate. To understand how health literacy and ethics are interrelated, the authors assess implant wearers’ perspectives on responsibilities and challenges when accessing and applying information and advice about the integration of (implant) technology in everyday life, as well as how individual skills, as well as a healthcare system’s responsiveness, impacts the dependence on a technological device. Though this research equally relates to individual health literacy, an important argument is made here regarding the organizational perspective: Healthcare organizations may not only allow information and technology to be medically comprehensible but acknowledge health literacy development as an inherently ethical task that requires consideration of an individuals’ “lifeworld” and the values attached to living with a technological device on a daily basis.

## 3. Conclusions

The purpose of this Special Issue was to better understand how to develop health literacy for a population with specific, often increased, health literacy requirements, such as people living with chronic diseases. Given this, it seems warranted to invite perspectives on a broad range of health literacy research objectives, contents, and formats. By “learning from international practices”, we hope to attract interest in and awareness of co-design methodologies, such as the use of the Ophelia process in different contexts, and of understanding dimensions of health literacy as perceived by people with lived experiences. Health literacy development is the ways in which health literacy promoting environments are created to enable people to access, understand, appraise, remember, and use information about health and health care, rather than putting the onus on individuals—especially those living in circumstances that perpetuate vulnerabilities—to always need to know how and be able to undertake the complex task of managing chronic disease. Ideally, the research findings in this Special Issue will provide a valuable addition to research or a change of perspective for those working in the field of health literacy. The articles reveal a clear argument for healthcare professionals, services, and researchers to dedicate resources and research priorities to actions that develop health literacy. Instead of expecting individuals to be responsible for navigating through an ever-growing and increasingly complex health information landscape, people living with chronic conditions will benefit from health literacy research that informs actions and policies that improve access to and use of health information and services.
