# Correction: Lander et al. Health Literacy Development among People with Chronic Diseases: Advancing the State of the Art and Learning from International Practices. *Int. J. Environ. Res. Public Health* 2022, *19*, 7315

**DOI:** 10.3390/ijerph20021510

**Published:** 2023-01-13

**Authors:** Jonas Lander, Marie-Luise Dierks, Melanie Hawkins

**Affiliations:** 1 Institute for Epidemiology, Social Medicine and Health Systems Research, Hannover Medical School, 30625 Hannover, Germany; 2 Centre for Global Health and Equity, Faculty of Health, Arts and Design, Department of Health and Biostatistics, Swinburne University of Technology, Melbourne, VIC 3122, Australia

The authors would like to make corrections to a recently published paper [1].

## Missing Funding

In the original publication, the funder German Research Foundation (Deutsche Forschungsgemeinschaft), DFG FOR 2959, project-id: 409800133; grant number: DI 1757/2-1 to authors Jonas Lander and Marie-Luise Dierks was not included. The authors apologize for any inconvenience caused and state that the scientific conclusions are unaffected. The original publication has also been updated.
